# Sensory Dysfunction in ALS and Other Motor Neuron Diseases: Clinical Relevance, Histopathology, Neurophysiology, and Insights from Neuroimaging

**DOI:** 10.3390/biomedicines13030559

**Published:** 2025-02-22

**Authors:** Jana Kleinerova, Rangariroyashe H. Chipika, Ee Ling Tan, Yana Yunusova, Véronique Marchand-Pauvert, Jan Kassubek, Pierre-Francois Pradat, Peter Bede

**Affiliations:** 1 Computational Neuroimaging Group, School of Medicine, Trinity College Dublin, D02 PN40 Dublin, Ireland; 2 Department of Speech-Language Pathology, University of Toronto, Toronto, ON M5S 1A1, Canada; 3 Laboratoire d’Imagerie Biomédicale, CNRS, INSERM, Sorbonne University, 75013 Paris, France; 4 Department of Neurology, University Hospital Ulm, 89081 Ulm, Germany; jan.kassubek@uni-ulm.de; 5 Department of Neurology, Pitié-Salpêtrière University Hospital, 75013 Paris, France; 6 Department of Neurology, St James’s Hospital Dublin, D08 NHY1 Dublin, Ireland

**Keywords:** amyotrophic lateral sclerosis, motor neuron disease, sensory, neuroimaging

## Abstract

**Background**: The clinical profiles of MNDs are dominated by inexorable motor decline, but subclinical proprioceptive, nociceptive and somatosensory deficits may also exacerbate mobility, dexterity, and bulbar function. While extra-motor pathology and frontotemporal involvement are widely recognised in motor neuron diseases (MNDs), reports of sensory involvement are conflicting. The potential contribution of sensory deficits to clinical disability is not firmly established and the spectrum of sensory manifestations is poorly characterised. **Methods**: A systematic review was conducted to examine the clinical, neuroimaging, electrophysiology and neuropathology evidence for sensory dysfunction in MND phenotypes. **Results**: In ALS, paraesthesia, pain, proprioceptive deficits and taste alterations are sporadically reported and there is also compelling electrophysiological, histological and imaging evidence of sensory network alterations. Gait impairment, impaired dexterity, and poor balance in ALS are likely to be multifactorial, with extrapyramidal, cerebellar, proprioceptive and vestibular deficits at play. Human imaging studies and animal models also confirm dorsal column-medial lemniscus pathway involvement as part of the disease process. Sensory symptoms are relatively common in spinal and bulbar muscular atrophy (SBMA) and Hereditary Spastic Paraplegia (HSP), but are inconsistently reported in primary lateral sclerosis (PLS) and in post-poliomyelitis syndrome (PPS). **Conclusions**: Establishing the prevalence and nature of sensory dysfunction across the spectrum of MNDs has a dual clinical and academic relevance. From a clinical perspective, subtle sensory deficits are likely to impact the disability profile and care needs of patients with MND. From an academic standpoint, sensory networks may be ideally suited to evaluate propagation patterns and the involvement of subcortical grey matter structures. Our review suggests that sensory dysfunction is an important albeit under-recognised facet of MND.

## 1. Introduction

### 1.1. The Clinical Spectrum of Motor Neuron Disease

Motor neuron diseases (MNDs) encompass a clinically and pathologically heterogeneous group of neurodegenerative disorders with distinct clinical, neuroimaging and biomarker profiles. Clinical phenotypes in MNDs are classically discussed along the spectrum of upper motor neuron (UMN) to lower motor neuron (LMN) dysfunction predominance including primary lateral sclerosis (PLS), hereditary spastic paraplegia (HSP), amyotrophic lateral sclerosis (ALS), spinal and bulbar muscular atrophy (SBMA or Kennedy’s disease) [1,2,3,4,5,6,7,8]. Another dimension of disease heterogeneity is the varying degree of frontotemporal dysfunction or comorbid frontotemporal dementia (FTD) in MNDs and phenotypes such as ALS-FTD, PLS-FTD, ALS with cognitive impairment (ALSci), and ALS with behavioural impairment (ALSbi) are also often distinguished [9,10]. Many neurologists would also regard HSP as a motor neuron disease [11,12,13], and low-incidence entities, such as monomelic ALS variants, O’Sullivan McLeod syndrome, and post-poliomyelitis syndrome are also often considered as MND subtypes [14,15,16,17,18,19,20,21,22,23].

### 1.2. Disease Heterogeneity and Extra-Motor Manifestations

Another facet of heterogeneity in MND is the relatively distinct clinical phenotypes associated with specific genetic variants which can often be linked to unique biomarker signatures [24,25,26,27,28,29]. Amyotrophic lateral sclerosis (ALS) is a neurodegenerative disease characterised by the concomitant degeneration of both the upper and lower motor neuron systems [30,31]. The core neuroimaging signature of ALS includes motor cortex, brainstem, corticospinal tract, and spinal cord degeneration, with subcortical grey matter degeneration also being observed [32]. It is increasingly recognised as a multi-system disorder, with extra-motor involvement being observed to be part of the disease process [31,33]. Pathology of sensory neurons in the dorsal root ganglia and sensory neuropathy are not widely acknowledged as part of the ALS syndrome [34], and there is a prevailing notion that ALS spares sensory networks [35], despite evidence of somatosensory disturbance that has been observed in ALS patients clinically [36,37,38,39], in electrophysiology [36,40,41,42,43], neuroimaging [41,44,45,46,47] and neuropathology [36,48,49,50,51,52] for several decades [31]. Postcentral neocortex involvement is regarded as a hallmark of “Stage 3” and thalamic involvement is regarded as an indicator of “Stage 2” of the Brettschneider--Braak pathological staging system proposed based on TDP-43 burden patterns [52,53]. From a clinical perspective, numbness [31], pain [54] and paraesthesia [37,55,56,57] are among some the most common sensory changes described by patients. While frank visual and auditory deficits are rarely observed, subtle changes in smell [58,59,60] and taste [61,62] are also occasionally reported. Proprioceptive deficits are also observed [63] and may have implications with regard to gait and dexterity. Additionally, sensory dysfunction may contribute to impaired cough reflex [64] and swallowing [65,66]. However, the findings of sensory pathology in ALS are conflicting with negative findings being reported in several studies [67,68,69,70,71,72,73]. Sensory disturbance is also observed in other motor neuron disease variants such as primary lateral sclerosis (PLS) [74,75,76]. The comprehensive characterisation of somatosensory pathophysiology in ALS is crucial for our academic understanding of disease heterogeneity, and findings of sensory pathology may have practical implications for rehabilitation efforts and the monitoring of disease progression. Furthermore, sensory dysfunction may have a significant impact on patients’ quality of life. The objective of this review is to investigate findings of sensory pathology in ALS and other MND phenotypes.

## 2. Methods

**Figure 1:**
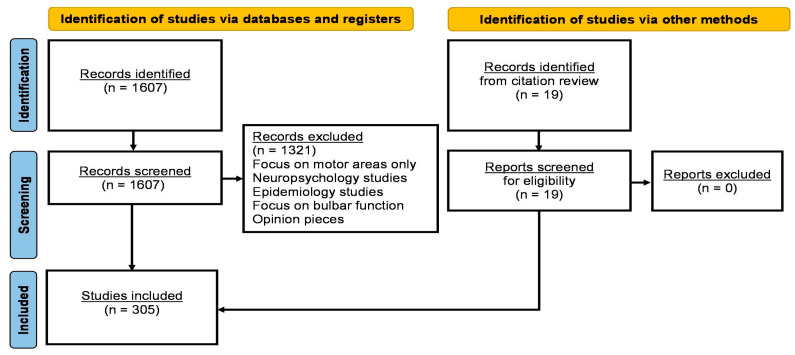
PRISMA Flow Chart.

A formal literature search was performed on PubMed using the core search terms “amyotrophic lateral sclerosis”, “motor neuron disease”, “primary lateral sclerosis”, “spinal and bulbar muscular atrophy”, “hereditary spastic paraplegia”, “kennedy’s disease”, and “post-polio syndrome” individually combined with each of the following keywords: “sensory”, “somatosensory”, “sensory cortex”, “postcentral gyrus”, “dorsal column”, “thalamus”, “proprioception”, “thermoception”, “nociception”, “bulbar sensation”, “taste”, “enjoyment of food”, “facial sensation”, “paraesthesia”, “electrophysiology”, “neurophysiology”, “magnetic resonance imaging”, “PET”, “spinal imaging”, “biopsy”, and “animal models”. Only original research papers specifically assessing somatosensory function were systematically reviewed. Review papers, meta-analyses, conference abstracts, opinion pieces and editorials were excluded. Where relevant, references of original research papers have were also reviewed. The review was conducted between August 2023 and October 2023 and only papers published in English were reviewed. Based on the above criteria, a total of 305 original research papers were selected and reviewed in accordance with the Preferred Reporting Items for Systematic Reviews and Meta-Analyses (PRISMA) 2020 recommendations (Figure 1).

## 3. Results

### 3.1. Clinical Observations

#### 3.1.1. Pain and Paraesthesia

Somatosensory symptoms and signs are well documented in ALS. According to one study, the most common symptom reported was numbness, followed by neuropathic pain, tingling, and reduced temperature sensation [31]. Sensory disturbance is more common in familial ALS than in sporadic ALS [30,77]. One study showed that 20% of familial ALS (fALS) cases manifest atypical features such as pain, paraesthesia or urgency micturition [55]. Pain is commonly reported in patients with ALS, with a study reporting a prevalence of 66% [54]. Pain was most commonly located in the neck and shoulders [54]. Neuropathic pain was reported in 9% of patients [54]. Pain intensity is not typically correlated with disease duration or physical disability [54]. Paraesthesia is often reported by patients [37,56,57], but it is also regarded as a potential side effect of Riluzole [78,79,80]. A small proportion of patients demonstrated sensory alterations on the quantitative sensory testing (QST) battery [49]. There are conflicting reports of impaired thermal sensory pathways. One study reports thermal threshold abnormalities [81], while another which investigated contact heat-evoked potentials in ALS patients proposed that the nociceptive pathway is not affected, suggesting that small fibres are spared in ALS [69].

#### 3.1.2. Gait and Balance Impairment

Gait impairment is a well-recognised facet of ALS. Despite a multitude of targeted studies [82,83,84], the substrates of impaired postural control and gait abnormalities are relatively challenging to untangle as proprioceptive, extrapyramidal, cerebellar and vestibular components are likely to contribute to these deficits [85,86,87,88,89]. Twenty-five percent of patients assessed in one study had proprioceptive impairments [63]. Nonetheless, abnormal postural reactions have been linked to impaired balance, and it has been proposed that impaired axial control leads to postural abnormalities [90] and impaired gait [91,92]. Extrapyramidal involvement is also suspected to contribute to stiffness and balance impairment in ALS [86,93]. Vestibular deficits have been consistently reported in ALS [88,93,94,95] and evaluated both from a diagnostic and management perspective. These, although they are under-recognised, are thought to increase the risk of falls [93]. Gait impairment and poor balance in ALS are likely to be multifactorial with extrapyramidal, cerebellar, proprioceptive and vestibular deficits at play; therefore, the assessment of the individual contribution of these deficits is very challenging [82,83,84,96].

#### 3.1.3. Gustatory, Olfactory, Pharyngeal and Laryngeal Manifestations

Other sensory manifestations such as a reduction in taste [62,97] have been reported, which may not only have negative quality of life implications, but the loss of enjoyment of eating may lead to reduced caloric intake. While taste and smell impairments [61] were reported by some studies, others detected no marked changes in olfaction and gustation [98], highlighting the need to assess these deficits prospectively in larger studies. It is noteworthy that several studies identified no abnormal findings in the sensory system [68,98,99], adding to the inconsistency in the literature and underlining the need for comprehensive studies to elucidate the degree and substrate of somatosensory dysfunction in MNDs. One study identified that 43.8% of patients with ALS reported taste alterations [100]. Changes in taste perception may have profound negative consequences on quality of life [62]. There is multimodal evidence of laryngeal sensory impairment in ALS. Laryngeal adduction reflex (LAR) abnormalities have been observed in as many as 20% of patients in some studies [101]. Laryngeal sensory changes are also commonly observed in the fibre-optic endoscopic evaluation of swallowing with sensory testing (FEEST) [65,66]. Thirty-three percent of patients with ALS had sensory deficits of the larynx in one study [65], while another detected deficits in as many as 54.5% of patients [66]. Sensory deficits are more commonly observed in bulbar-onset ALS patients [65] and laryngeal and pharyngeal sensory deficits may contribute to dysphagia [102,103,104]. Olfactory impairment has also been observed [58,59,60]. One study showed that changes in respiratory function correlate with deficits in olfaction [60]. Cutaneous sensory and autonomic denervation has been reported in both ALS and PLS, but the pathophysiological mechanisms behind these changes are not well characterised [50]. While the discussion and review of autonomic dysfunction in MNDs is beyond the scope of this paper, autonomic dysfunction has been consistently reported in both sporadic and familial forms of ALS [105,106,107,108,109,110,111,112]) (Table 1).

#### 3.1.4. Insights from Electrophysiology Studies

Clinical reports of sensory involvement are increasingly complemented by neurophysiology studies (Table 2). The incidence of sensory nerve conduction abnormalities in ALS varies considerably from study to study [121,122] from as low as 14.7% [123] to as high as 66.7% [121]. While a study showed that 22.7% of patients with ALS had sensory abnormalities in at least one nerve [42], another study of 154 patients found that abnormal sensory nerve conduction is only detected in a minority of ALS patients [124]. It has been consistently shown that patients with ALS have a slower sensory conduction velocity [125,126] and it has been suggested that sensory involvement is more common in *C9orf72* hexanucleotide repeat expansion carriers. Electrophysiological evidence of a sensory neuropathy was observed in 38% of *C9orf72* positive patients compared to 21% of *C9orf72*-negative ALS patients [127]. Sensory deficits are also commonly seen in *SOD1* patients [110], and in general, sensory disturbance is more commonly observed in familial ALS patients [109,118]. A comprehensive review of electrodiagnostic tests in ALS confirmed sensory signs in 32% of patients, and in 27% of patients, sural SNAPs were abnormal [36]. Reduced conduction velocity [43] and abnormal sensory nerve action potentials (SNAPs) are commonly observed [43,124,128], and one study showed that SNAP amplitude deteriorated with disease progression, although it remained within the normal range [39]. SNAP has been proposed as a prognostic indicator, as one study showed a superior prognosis in those with lower median nerve SNAP amplitudes, but only in patients younger than 57 years old [129]. It has also been suggested that compound muscle action potential (CMAP) and SNAP amplitudes of the median nerve are independent prognostic factors of sporadic ALS [129]. Abnormal somatosensory evoked potentials (SEPs) were previously used to exclude a diagnosis of ALS [130]; however, abnormal SEPs are commonly observed in both ALS [40,131,132,133,134,135,136] and PLS [135]. Abnormal median and tibial nerve SEPs have been reported in ALS [134]. A study showed sensory action potential amplitude (SAPa) reductions in 22% of patients, affecting the median, ulnar and sural nerves [137]. Most studies are consistent in confirming that sensory nerve conduction measures are more likely to capture pathological change than conventional sensory measures [121]. One study identified that 60% of patients had abnormal findings on sensory testing, but suggested that the changes may be non-progressive [126]. Despite of a plethora of studies reporting abnormal sensory measures in ALS, there are a handful of studies emphasising the absence of sensory findings in ALS [70,73,138,139,140]. This apparent inconsistency is probably best resolved by large, prospective multi-centre studies applying a methodologically standardised protocol. From a biomarker perspective, sensory cortex hyperexcitability was linked to shorter survival [132]. Several studies seem to indicate that abnormal SEPs and SNAPs often do not manifest in clinical signs of complaints, suggesting that sensory abnormalities may often remain subclinical [39,141]. A-beta or A-delta sensory fibres, and in some cases both, are shown to be impaired in ALS [133]. There is a relative scarcity of longitudinal neurophysiology studies focusing on sensory involvement.

#### 3.1.5. Histopathology and Animal Model Data

pTDP-43 pathology has been consistently detected in the somatosensory cortex [162,163,164,165] as well as the thalamus [52,53]. Anatomical pTDP-43 burden patterns were used in the development of the Brettschneider--Braak pathological staging system in ALS [52,53], which has since been extensively validated by neuroimaging studies [166,167,168,169,170]. Postcentral neocortex involvement is regarded as a hallmark of “Stage 3” [52,53]. Studies also capture the pathology of dorsal root axons and dorsal root ganglion (DRG) cell bodies [171]. Thalamic pathology in ALS also has been extensively studied. Thalamic involvement is regarded as an indicator of “Stage 2” of the pTDP-43 staging system [52,53]. Post-mortem studies have consistently commented on both global thalamic degeneration in ALS [172] as well as the predilection to specific nuclei [173,174]. A study investigating post-mortem brains using 7T MRI as well as histopathology describes iron deposition in the thalamus [175]. Considerable thalamic dipeptide protein repeat (DPR) [176] and moderate p62 [177] burden were identified in individuals carrying the *C9orf72* hexanucleotide repeat expansions. An interesting pathology report of seven patients with ALS who were in a locked-in state described considerable somatosensory, auditory, and gustatory pathway involvement with the relative preservation of visual and olfactory pathways [178]. Loss of neurons in the dorsal root ganglia as well as degeneration of posterior columns can be detected both ante and post mortem [73,179]. Contrary to brain and spinal cord reports, peripheral nervous system findings are somewhat inconsistent. Intra-epidermal nerve fibre density has been found to be normal in some studies [114] and reduced in others [48,51]. Both large-calibre and small-calibre sensory fibres are thought to be affected in ALS [36] and the sural nerve has been consistently shown to be affected [180,181,182]. Inflammatory cell infiltrates [183], reduced myelin thickness [184], and axonal loss [182] have all been observed in the sural nerve. Pathological change was detected in 91% of patients who underwent sural nerve biopsy, and large-calibre myelinated fibres may be particularly vulnerable [36]. A reduction in myelinated fibres was also observed in the peroneal nerve [56,185]. Small-fibre neuropathy is also thought to be a feature of ALS. It was demonstrated by a study [51] showing a significant epidermal small fibre density reduction in the distal calf. Laryngeal dysfunction is a cardinal feature of ALS [104] and the sensory components of laryngeal dysfunction have been specifically investigated in dedicated multimodal studies [65,101]. Aberrant or absent intraepidermal fibres were noted on laryngeal biopsies [65]. While murine models of ALS are not universally regarded as representative of the complex pathobiology of human ALS, TDP43 animal models have also consistently shown sensory pathology [186,187]. *SOD1* animal models revealed pathology in central sensory regions [188,189,190], DRG neurons [30,33], DRG axons [191] and sensory neurons [192,193]. Wallerian degeneration of sensory nerves is readily observed in *SOD1* mouse models [194]. A diffusion tensor imaging (DTI) study of an *SOD1* animal model also confirmed sensory involvement in the symptomatic disease phase [188]. Other animal studies, however, revealed that sensory white matter fibres were preserved [72,195] and similarly, sensory deficits were not observed by other studies [72,195,196]. Histological data pertaining to somatosensory pathology are summarised in Table 3.

#### 3.1.6. Neuroimaging

**Figure 2:**
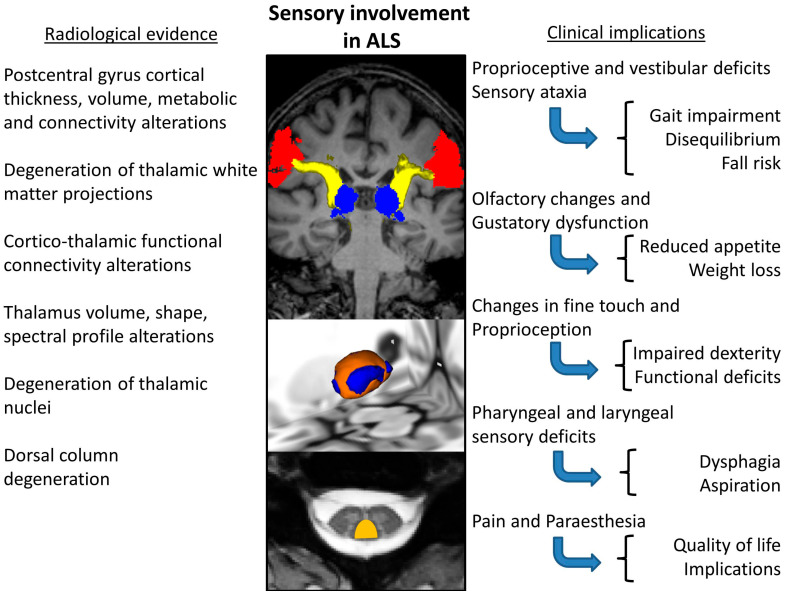
Consensus of imaging findings pertaining to sensory dysfunction in ALS and a summary of putative clinical ramifications.

Neuroimaging offers a wealth of evidence for somatosensory involvement in ALS (Table 4.). With the advent of spinal imaging in ALS [204,205], dorsal column degeneration [41,179,206] has been consistently demonstrated by neuroimaging studies [41,179,206]. One study reported a significant correlation between abnormal DTI measures of sensory fibres and N9 amplitude [41]. Combining spinal imaging and neurophysiology has shown sub-clinical deficits of the sensory system in up to 85% of ALS patients [41]. Dorsal column changes are observed soon after symptom onset; therefore, it is possible that sensory involvement is grossly underestimated as an early feature of ALS [41,179]. One study investigated sensory pathway dysfunction in patients with ALS, using a combination of diffusion tensor imaging (DTI), magnetization transfer and atrophy index, demonstrating considerable dorsal column pathology within a year of symptom onset [179]. Thalamic pathology has been extensively studied in the literature of ALS [207,208] and a multitude of segmentation techniques have been implemented to demonstrate focal thalamic involvement affecting specific nuclei projecting to specific cortical [32,47,207,208,209] and limbic regions [209,210]. Thalamic atrophy is particularly significant in ALS-FTD [10,174,209,211,212,213]. Thalamic regions mediating somatosensory circuits are involved in both *C9orf72*-negative and -positive ALS patients [47]. Thalamus pathology [209,214,215,216,217,218,219,220,221,222,223,224,225,226,227,228,229] has not only been demonstrated on structural [230,231,232] imaging, but also using diffusion tensor imaging (DTI) [233], functional MRI (fMRI) [46], PET [217,220], and magnetic resonance spectroscopy (MRS) [234,235]. Structural imaging studies are not only consistent in demonstrating postcentral gyrus atrophy [44,45,47,230,231,236,237,238], but insular, superior temporal, transverse temporal, supramarginal, and lateral occipital cortical thickness reductions have also been reported, which are thought to be more marked in hexanucleotide repeat expansion carriers in *C9orf72* [47]. In addition to cortical thickness analyses, functional [239,240], morphometric [241], susceptibility [242], spectroscopy [243] and diffusion studies [244] have all demonstrated parietal and occipital pathology in ALS. Functional imaging consistently reveals changes in the somatosensory cortex [245]. Reduced right regional coherence in the postcentral gyrus was seen to correlate with high disease severity, while increased regional coherence in the left postcentral gyrus was associated with longer disease duration [236]. The reduced fractional amplitude of low-frequency fluctuations (fALFF) was also observed in the right postcentral gyrus [238]. A reduced Na/Cr resonance intensity ratio has been demonstrated on spectroscopy in the postcentral gyrus [235]. The quantitative analyses of the integrity of white matter tracts involved in somatosensory processing revealed medial lemniscus posterior thalamic radiation diffusivity alterations in patients with ALS [47]. In summary, neuroimaging offers ample evidence of the degeneration of key cortical, subcortical, thalamic, spinal and white matter components of somatosensory networks in ALS. Consensus imaging findings pertaining to sensory dysfunction in ALS as summarised in Figure 2.

### 3.2. Other Motor Neuron Diseases

Reports of sensory alterations in primary lateral sclerosis (PLS) are inconsistent, and most studies focus on precentral gyrus, corticospinal tract and brainstem degeneration [271,272,273]. More recent studies, however, have demonstrated considerable extra-motor and thalamic pathologies in PLS [274,275]. Increased functional connectivity within the sensorimotor network was observed in patients with a faster progression and greater disability [74]. Thalamic volume reductions and shape deformations have been captured by most PLS studies evaluating this structure [274,276,277]. While the cortical thickness of primary sensory cortex is not reduced in PLS [275,278], some postcentral gyrus grey matter density reductions may be observed on morphometric analyses [275]. While Kennedy’s disease or spinal and bulbar muscular atrophy (SBMA) is primarily regarded as a lower motor neuron disease [1,5], widespread frontal and parietal degeneration has been highlighted by some imaging studies [10,279,280]. Imaging studies of SBMA have also consistently captured cerebellar [279,281], brainstem [279,282] and limbic [279,281] pathologies highlighting that the central nervous system is involved. In SBMA, reduced [75,76,283,284,285,286,287,288,289] or absent [290] SNAP has been consistently observed and SNAP does not appear to be correlated to CAG repeat size [291]. Axonal degeneration and the loss of myelinated nerve fibres were observed in the sural nerve [292]. Altered visual and auditory EPs [293], decreased somatosensory EPs [293] and prolonged somatosensory-evoked responses [294] were also observed. Post-polio syndrome (PPS) is a rare entity that affects poliomyelitis survivors decades after their initial infection. It typically presents after a long period of neurological stability and may manifest in myalgia, limb fatigability, new-onset muscle weakness, muscle bulk loss, and often as generalised fatigue [20,295]. While some post mortem studies have suggested a predilection to thalamic and hypothalamic involvement following poliomyelitis [296] and widespread cerebral involvement [297,298,299,300], other studies emphasise the absence of CNS involvement [301]. Abnormal SEPs have been observed in ageing polio survivors [302], but imaging studies of patients with post-polio syndrome are inconsistent. While early imaging studies have emphasised a considerable white matter hyperintensity burden in the reticular formation and medial lemniscus [296], subsequent quantitative imaging studies have not identified significant postcentral gyrus, thalamic or white matter alterations [21,303]. Hereditary spastic paraplegia (HSP) is a genetically and clinically heterogeneous group of neurodegenerative disorders typically divided into ‘pure’ and ‘complicated’ forms. In addition to spastic paraparesis, complicated HSP (cHSP), may be associated with cerebellar, extrapyramidal, optic nerve, cognitive impairment, and sensory manifestations. Imaging studies have described genotype-specific patterns of cerebral atrophy often including somatosensory regions [12]. Postcentral cortical thinning has been specifically highlighted in SPG4 [304] and SPG8 [305]. Morphometric [306,307,308,309], PET [310,311,312], SPECT [313] and spectroscopy [314] studies have consistently detected thalamic pathology in various HSP genotypes. Diffusion tensor imaging has identified changes in white matter integrity in thalamic radiations [315].

## 4. Discussion

Somatosensory involvement is an overlooked aspect of motor neuron diseases despite its likely contribution to bulbar dysfunction, impaired dexterity and gait impairment [82,83,84]. While subtle sensory symptoms are often reported by patients, in the face of relentless motor decline, targeted clinical or neurophysiological examination is seldom performed to specifically assess sensory dysfunction in routine clinical care. Clinical care is centred on the most pressing clinical aspects of ALS, such as respiratory and bulbar involvement and the maintenance of motor independence. Nonetheless, raising awareness of sensory involvement and the integration of clinical, imaging and neurophysiological evidence with regard to sensory involvement is crucial both from a clinical and an academic perspective.

From a clinical standpoint, the detection of proprioceptive deficits, addressing pain and the consideration of the sensory aspects of bulbar dysfunction, gait impairment and changes in dexterity have considerable practical relevance (Figure 2). It is also conceivable that in ALS, deficits in sensorimotor integration may contribute to impaired dexterity. ALS patients exhibit poor performance on the nine-hole peg test (NHPT) which has a moderate negative correlation with ALSFRS-R handwriting scores [316]. Impaired dexterity in ALS is multifactorial, and a combination of UMN, LMN, extrapyramidal, cerebellar and sensory components are likely at play. These deficits have a significant impact on independence, affecting writing, typing, driving, and getting dressed, among other essential daily tasks. Gait impairment in ALS is also thought to be multifactorial, encompassing extrapyramidal [84], cerebellar [87], postural [90] and vestibular [93] components in addition to primary motor system degeneration. The exact degree to which sensory afferent dysfunction contributes to gait impairment in ALS is very challenging to assess clinically due to extensive lower motor neuron and pyramidal involvement. Despite the compelling evidence of considerable sensory dysfunction in ALS, current rehabilitation strategies in ALS focus nearly exclusively on motor dysfunction and spasticity. The recognition of proprioceptive and vestibular deficits may guide fall prevention strategies [93]. The underpinnings of impaired dexterity are seldom evaluated comprehensively [316,317] and often are merely attributed to upper and lower motor neuron dysfunction overlooking proprioceptive, cerebellar and extrapyramidal components. Patients with ALS need to interact confidently with communication aids, take their medications, put on their NIV masks, and rely increasingly on their phones, tablets and other electronic devices; therefore, dexterity is a crucial aspect of their condition and the maintenance of function is the mainstay of occupational therapy [318,319]. Sensory dysfunction is also likely to contribute to bulbar dysfunction. Pharyngeal sensory deficits are thought to be more common in bulbar-onset ALS as evidenced by the endoscopic evaluation of swallowing with sensory testing [65,66]. ALS patients also report increased sensitivity to an upper airway irritant [64]. Evidence from Parkinson’s disease suggests that somatosensory deficits can also contribute to dysarthria [320]. Progressive dysphagia and dysarthria are often solely attributed to muscle weakness, overlooking the potential contribution of sensory impairment. Weight-loss and decreased oral intake are hallmarks of bulbar-onset ALS and are typically attributed to progressive dysphagia, metabolic and endocrine changes and apathy. Subtle changes in taste, impaired olfaction and the resulting reduced enjoyment of food are less commonly considered [58,59,60,61,62,321]. While some degree of limb paraesthesia is often experienced in ALS, clinicians rarely ask about sensory symptoms in established ALS cases.

Genotype-associated sensory profiles are difficult to establish based on the available literature. The majority of ALS studies are not stratified for genetic status, do not provide comprehensive screening for genetic variants, or only screen for a panel of the most common genetic variants associated with the disease (*SOD1*, *C9orf72*, *TARDBP*, *FUS,* etc.). There is therefore a paucity of evidence to link sensory-predominant manifestations to specific genotypes. One of the best characterised ALS cohorts is patients carting the GGGGCC hexanucleotide repeat expansion in *C9orf72*, a genotype that is typically linked to extensive extra-motor, subcortical, thalamic and cerebellar manifestations [25,27,322] and pre-symptomatic thalamic changes [28,323]. It is noteworthy, however, that extensive subcortical and frontotemporal change is not unique to the *C9orf72* genotype [324]. Existing epidemiology studies of ALS focus on genotype-associated survival profiles, neuropsychological traits, longitudinal trajectories and the rate of decline [325], but sensory aspects are often overlooked. Accordingly, future studies that are clinical or radiological should meticulously screen their patients for ALS-associated genetic variants and specifically examine whether sensory manifestations are more common in a certain genotype.

From an academic standpoint, considerable research gaps persist and there is a notable shortage of recent papers evaluating sensory and somatosensory dysfunction in ALS and other MNDs. Despite the practical clinical implications of sensory deficits in ALS, many of the original studies we identified are a few years old. There is a sense that instead of pursuing classical clinico-radiological descriptive studies, there may be a trend in pursuing more “topical” facets of ALS research more recently, such as antisense oligonucleotide (ASO) development, machine learning (ML), artificial intelligence (AI) application, cluster analyses, presymptomatic studies, and assistive and wearable technologies [326,327,328,329,330,331,332]. As outlined in this review, there is ample clinical, imaging, histopathology and neurophysiology evidence that the somatosensory system is not spared in ALS. Despite the considerable amount of the literature, however, the majority of reports are from cross-sectional, single-timepoint studies. It is therefore challenging to determine whether sensory pathology is an early or late feature of the disease. There are sporadic reports of sensory involvement preceding motor pathology [38,333] and there are reports of thalamic pathology preceding cortical involvement in presymptomatic mutation carriers [28,334]. However, there is a notable scarcity of multi-timepoint, longitudinal studies evaluating progressive changes [335,336,337,338] and there is a particular paucity of studies specifically examining the evolution of somatosensory dysfunction. Few studies assess sensory dysfunction comprehensively using multiple complementary methods. Certain sensory domains such as visual, auditory, olfactory and gustatory deficits are particularly commonly overlooked [58,59,60,61,62]. Abnormal visual evoked potentials [339,340], retinal changes on optical coherence tomography (OCT) [341,342] and changes in auditory evoked potentials [40,340] have been consistently demonstrated in ALS. Among a wide range of neuropsychological domains affected in ALS [210,343,344,345], impaired visuospatial ability has been consistently reported in a proportion of patients [110,346]. Pathology in the lateral posterior nucleus of the thalamus, which plays a role in visual saliency and visually guided behaviours with afferents from the superior colliculus, primary visual, auditory and somatosensory cortices and efferents to the parietal association cortex, has also been observed [207]. Visuospatial dysfunction may negatively impact basic daily activities such as ambulation, driving and reading. Subclinical auditory deficits may also have considerable ramifications. Impaired taste and smell may have unrecognised quality-of-life implications as well as impacts on patients’ appetites [62]. While the discussion of therapeutic advances is beyond the scope of this paper, our review highlights the considerable clinical, radiological and genetic heterogeneity of motor neuron diseases, and supports the notion that instead of pursuing the development of a single disease-modifying drug that is effective in all phenotypes, a precision medicine strategy is required which would work in specific genotypes and phenotypes. The transition in drug development from generic “neuroprotective” approaches to precision therapies is well demonstrated by recent antisense oligonucleotide trials [347,348,349].

## 5. Conclusions

ALS should no longer be considered a condition that exclusive involves motor and frontotemporal regions. Sensory alterations are likely to contribute to some of the core symptoms of ALS, including bulbar dysfunction, gait impairment, and impaired dexterity. Despite the wealth of clinical, neurophysiology, imaging, and histopathology data, sensory deficits remain glaringly under-evaluated in ALS and other motor neuron diseases. The recognition and routine assessment of the sensory system is crucial for precision clinical evaluation, disease monitoring and the understanding of disease biology.

## Figures and Tables

**Table 1:** Clinical evidence for somatosensory impairment in ALS.

Author and Year of Publication	Study Participants	Method of Assessment	Significant Findings
Tabor-Gray et al., 2021 [64]	32 with ALS, 34 healthy controls	Sensory patients rated urge to cough (UtC), cough spirometry metrics	- ALS patients show increased sensitivity to an upper airway irritant - ALS patients have slower and weaker expiratory cough motor output
Krieg et al., 2019 [90]	12 with ALS, 12 healthy controls	Spontaneous sway measures, measures of postural reactions	- Spontaneous sway amplitudes and velocities significantly larger in patients with ALS - Sway frequencies higher in patients with ALS - High correlations between abnormal postural reactions and motor or balance deficits
Simmatis et al., 2019 [63]	17 with ALS	Robot-based sensorimotor, cognitive and proprioceptive performance	- 20–69% displayed sensorimotor impairments - 25% have proprioceptive impairments
Tarlarini et al., 2019 [62]	32 with ALS	Taste observations	- Perceived reduction in taste in ALS - Loss of taste negatively related to QoL
Gunther et al., 2018 [60]	94 with ALS, 81 healthy controls	Olfactory performance-Sniffin Sticks	- Impairment of olfaction detected in patients
Lopes et al., 2018 [113]	80 with MND, 32 healthy controls	QST, CPM, clinical	- Pain frequently reported
Isak et al., 2017 [49]	32 with ALS, 32 healthy controls	QST and IENFD skin biopsy	- Mean values of QST and IENFD in normal range
Marjanovic et al., 2017 [110]	241 with ALS	Clinical	- Sensory and sphincter disturbances dominant in patients with *SOD1 L114F* - Significant sensory involvement in *SOD1 D90A*
Gunther et al., 2016 [61]	90 with MND, 96 healthy controls	NMS questionnaire	- Impaired taste and smell observed in MND
Moisset et al., 2016 [54]	96 with ALS	Pain assessment	- 66% of patients report pain, 9% of patients report neuropathic pain
Ruoppolo et al., 2016 [65]	114 with ALS	evaluation of swallowing, including a fibre-optic endoscopic evaluation of swallowing with sensory testing and biopsy	- 38 patients (33%) had sensory deficit of the larynx - Sensory deficit of the larynx was more common in bulbar-onset ALS
Truini et al., 2015 [114]	24 with ALS	SNAP, NCV, QST	- Normal thermal-pain perceptive thresholds
Ferrari et al., 2014 [115]	8 with ALS, 7 healthy controls	Corneal confocal microscopy	- Reduced corneal small-fibre sensory nerve number and branching in patients with ALS
Radovanovic et al., 2014 [91]	27 with ALS, 29 healthy controls	Simple walking test, dual-motor task, dual-mental task, combined motor and mental tasks	- Impaired gait in dual tasks in spinal and bulbar onset ALS
Sanjak et al., 2014 [93]	19 with ALS, 15 healthy controls	Clinical	- Nearly 37% of ambulatory ALS with normal clinical balance testing have reduced ability to use vestibular input
Hineno et al., 2012 [109]	24 with ALS	Clinical, neurophysiology, neuropathology	- Half of patients had neurogenic bladder and sensory impairment
Jesus et al., 2012 [100]	40 ALS	Clinical	- 43.8% of patients report taste disorders
Lang et al., 2011 [98]	26 with ALS, 26 healthy controls	Olfactory performance- Sniffin Sticks	- No difference in ability
Pradat et al., 2009 [86]	39 with ALS	Clinical	- Extrapyramidal involvement plays role in stiffness and balance impairment in some ALS patients
Liao et al., 2008 [92]	13 with ALS, 51 healthy controls	Gait assymetry	- Gait symmetry significantly disturbed
Hammad et al., 2007 [36]	103 with ALS	SNAP, biopsy	- Sensory symptoms/signs seen in 32% of patients with ALS
Amin et al., 2006 [66]	22 with ALS	Laryngeal sensation	- Abnormal sensation in 54.5% of patients with ALS - Sensory deficits in the larynx present in ALS
Deepika et al., 2006 [68]	20 with ALS, 20 healthy controls	QST	- Normal thermal thresholds on QST
Khoris et al., 2000 [57]	NA	Genetics	- Paraesthesia commonly reported
Camu et al., 1999 [55]	109 with ALS	Clinical observations	- Pain, paraesthesia and urgency micturition experienced
Hawkes et al., 1998 [59]	58 with MND, 135 healthy controls	UPSIT, OEP	- Smell identification slightly worse overall in MND - Bulbar patients scored significantly less on UPSIT
Mogyoros et al., 1998 [99]	21 with ALS, 14 healthy controls	Paraesthesia due to ischemic compression	- Patients experience less or no paraesthesia during and after release of ischemic compression
Rothstein et al., 1992 [116]	13 with ALS, 15 with Alzheimer’s disease, 12 with Huntington’s, 17 healthy controls	Glutamate transport	- Reduced maximum velocity of transport of high-affinity glutamate uptake in synaptosomes from somatosensory cortex
Wirguin et al., 1992 [117]	31 with ALS	Clinical	- Normal sensory conduction
Elian et al., 1991 [58]	15 with MND, 10 healthy controls	UPSIT	- Significant olfactory impairment in MND
Li et al., 1988 [77]	NA	Clinical	- More frequent occurrence of sensory symptoms at presentation in familial cases (15%) than sporadic cases (5%)
Gubbay et al., 1985 [37]	318 with ALS	Clinical observations	- Sphincter disturbance and deep sensation loss observed
Jamal et al., 1985 [81]	40 with MND, 40 healthy controls	Heat threshold and cold threshold	- Abnormal thermal thresholds in 80% of MND patients
Hamida et al., 1984 [118]	102 with ALS	Clinical	- Juvenile form associated with sensory symptoms
Jokelainen et al., 1977 [119]	255 with ALS	Clinical	- Sensory disturbances absent, sphincter abnormalities reported in 3 patients
Alter et al., 1976 [120]	14 with ALS	Clinical	- Peripheral sensory impairment observed in one family

**Table 2:** Neurophysiology evidence of sensory impairment in MNDs.

Author and Year of Publication	Study Participants	Methodology	Significant Findings
Harada et al., 2021 [142]	23 with ALS, 14 healthy controls	SEP	- Pain-related SEP amplitudes significantly lower in ALS - No significant differences in pain-related SEP parameters between patients with or without sensory symptoms
Norioka et al., 2021 [143]	145 with ALS, 57 healthy controls	SEP	- ALS demonstrated larger N20o-p, N20p-P25p, and early and late HFO amplitudes
Imai et al., 2020 [129]	190 with ALS	SNAP	- SNAP identified as a prognostic factor of sporadic ALS
Nardone et al., 2020 [144]	10 with ALS, 10 healthy controls	SEP	- Large amplitude reduction in post-synaptic HF-SEP burst in patients with disease duration > 2 yrs
Hoffken et al., 2019 [145]	15 with ALS, 15 healthy controls	SEP	- Disinhibition of somatosensory cortex
Liu et al., 2019 [123]	150 with ALS	EMG	- Sensory system damaged in 22 patients on nerve conduction
Pegat et al., 2019 [127]	53 with ALS	Clinical and electrodiagnostic data	- 38% with C9+ ALS and 21% with C9− ALS have electrophysiological sensory neuropathy
Matamala et al., 2018 [146]	28 with ALS, 20 healthy controls	MUNE, SNAP	- Normal axonal membrane properties in myelinated sensory axons
Sangari et al., 2018 [131]	21 with ALS, 21 healthy controls	SEPs	- Late cortical components more depressed than early ones
Shimizu et al., 2018 [132]	145 with ALS, 73 healthy controls	SEP (N13, N20, P25)	- Larger amplitude of N20p-P25p in the median nerve SEP - Sensory cortex hyperexcitability predicts short survival
Kulkantrakorn et al., 2017 [122]	25 with ALS, 11 with PMA, 1 with PLS, 1 with Kennedy disease	NCS	- Sensory studies abnormal in 16.3%
Dalla Bella et al., 2016 [48]	57 with ALS	Sural nerve conduction study	- Sural nerve conduction study abnormal in 2 patients
Isak et al., 2016 [133]	18 with ALS, 31 healthy controls	LEPs, SSEPs	- Longer N2 and P2 latencies, smaller N2P2 LEPs; abnormal LEPs in 72.2% of patients with ALS - Longer latencies for median and tibial SSEPs; abnormal SSEPs in 56.6%
Ren et al., 2016 [124]	154 with ALS	SCV, SNAP	- Reduced sensory conduction velocity in 1.22–2.73% - Reduced SNAP amplitude in 0–1.82% and sural nerve SNAP absent in 0–1.22% of patients with ALS
Iglesias et al., 2015 [41]	21 with ALS, 21 healthy controls	SEPs	- Altered SEPs correlated with disease duration - Spinal imaging and electrophysiology together identify ~85% of patients with subclinical sensory defect
Jin et al., 2014 [147]	97 with ALS, 100 with Hirayama disease, 32 with cervical spondylotic amyotrophy	SNAP, conduction velocity	- Conduction velocity of sensory nerve and amplitude of SNAP in unaffected limb were normal
Hineno et al., 2012 [109]	24 with ALS	Neurophysiology, clinical	- Half the patients have neurogenic bladder and sensory impairment
Simone et al., 2010 [148]	24 with ALS, 23 healthy controls	LEPs	- LEP abnormalities present but no correlation with pain intensity or other clinical features
Xu et al., 2009 [69]	60 with ALS, 60 healthy controls	Contact heat-evoked potentials	- No significant differences in CHEP, suggesting that nociceptive pathway is intact
Pugdahl et al., 2008 [43]	35 with ALS, 35 healthy controls	SNAP	- Reduced SNAP amplitude or reduced conduction velocity or both found in 6 ALS patients
Hamada et al., 2007 [149]	26 with ALS, 15 healthy controls	SEPs, CMAP	- Altered median nerve SEP amplitude associated with motor disturbances - Central sensory conduction time and N20 duration prolonged
Hammad et al., 2007 [36]	103 with ALS	SNAP, biopsy	- Sural SNAP amplitudes abnormal in 27%, pathologic abnormalities in 91%
Pugdahl et al., 2007 [42]	88 with ALS	SCNV, SNAP	- 20 patients had sensory NCS abnormalities in at least one nerve - Of these, 11 had electrophysiological polyneuropathy
Argyriou et al., 2006 [70]	23 with ALS, 23 healthy controls	Sensory conduction study	- Sensory conduction study was normal
Koszewicz et al., 2005 [138]	19 with ALS, 20 healthy controls	Sensory conduction study	- Sensory conduction parameters do not differ significantly between groups
Ogata et al., 2001 [150]	12 with ALS	SEP	- Abnormal posterior tibial nerve and median nerve SEPs detected in some patients
de Carvalho et al., 2000 [139]	70 with ALS, 35 healthy controls	CMAP, CV, sensory potentials	- No conduction block, and sensory potentials were normal
Matsumoto et al., 1999 [151]	14 with ALS	SSCV, SSEP	- Degree of reduction in SCCVs correlated with degree of reduction in vibration sense and duration of illness
Rabin et al., 1999 [73]	49 with ALS	Electrodiagnostic	- Sensory conduction studies and quantitative sensory testing were normal
Schulte-Mattler et al., 1999 [152]	23 with ALS, 23 healthy controls	SNCV, SNAPA	- Median sensory NCV abnormally reduced in 3 patients
Theys et al., 1999 [126]	50 with ALS, 20 healthy controls	Motor and sensory assessment	- NCS and SEP showed abnormal delay in peripheral and central sensory pathways - At least one sensory test abnormal in 60% of patients - Significant decrease in amplitude of SNAPs of sural nerves over 6 month follow-up
Theys et al., 1999 [126]	50 with ALS	SEP, Thermal thresholds	- Abnormal sensory tests in 60% of the patients
Emeryk-Szajewska et al.,1998 [153]	94 with ALS, 2 with PLS, 3 with primary bulbar palsy, 6 with primary motor spinal atrophy	Nerve conduction	- Slowing of conduction velocity in 25% of sensory fibres in median nerve, 11% of sural nerve
Mogyoros et al., 1998 [154]	23 with ALS, 32 healthy controls	CMAP, CSAP	- Strength duration time constant of sensory fibres declines with age; no difference between patients and controls
Georgesco et al., 1997 [155]	24 with ALS, 17 healthy controls	SEP	- Alterations in SEPs’ cortical components of all lower limb nerves
Zanette et al., 1996 [156]	29 with ALS, 10 with PMA	SEP	- SEPs altered in 22 ALS patients, but unaffected in 10 PMA patients
Georgesco et al., 1994 [135]	21 with ALS, 7 with PLS	SEPs	- SEPs abnormal in ALS and PLS
Constantinovici et al., 1993 [136]	10 with ALS	SEPs	- 9 patients with abnormal parietal SEPs to tibial nerve stimulation
Gregory et al., 1993 [39]	19 with ALS, 12 healthy controls	SNAP, SNCV	- Significant fall in amplitude of SNAPs in medial, radial and sural nerve - SNCV not altered
Lukomski et al., 1993 [94]	18 with ALS	ENG exam	- 8 patients have lesion of central part of vestibular system, 2 have peripheral vestibular disturbance
Mondelli et al., 1993 [137]	64 with ALS, 60 healthy controls	MCV, SCV, SAPa	- SAPas more affected than SCVs - Parallel decrease in SCVs and MCVs over time in 14 patients
Palma et al., 1993 [157]	NA	BAEP, VEP, SEP	- No difference in BAEP and VEP - Reduction in N13 amplitude and P22 latency in SEP recordings
Behnia et al., 1991 [128]	133 with ALS	SNAP	- SNAP amplitudes maybe abnormal in a small proportion of ALS patients
Gao et al., 1991 [158]	343 with MND	Sensory nerve conduction velocities	- Sensory nerve conduction velocities were almost always normal
Shefner et al., 1991 [125]	18 with ALS	Compound sensory action potentials	- 18 patients had abnormally reduced minimum conduction velocity
Subramaniam et al., 1990 [134]	27 with MND	SEP, PSVEP, BAEP	- Median nerve SEPs abnormal in 8 out of 27 patients with MND and tibial nerve SEPs abnormal in 3 out of 21 patients with MND
Zanette et al., 1990 [159]	26 with MND, 20 healthy controls	SEPs	- Central conduction time was abnormal in 3 patients
Facco et al., 1989 [160]	19 with ALS	SEP	- N9-N13 significantly delayed, N13-N20 normal
Berardelli et al., 1987 [140]	11 with MND, 20 healthy controls	SEP, MCV	- Somatosensory responses from wrist stimulation were normal
Radtke et al., 1986 [40]	17 with ALS	Sensory evoked potential	- SEPs abnormal in 7 out of 16 patients after lower-extremity stimulation and 2 out of 16 patients after upper-extremity stimulation
Cosi et al., 1984 [161]	45 with ALS	SEPs	- Decreased amplitude of SEPs from tibial nerve

**Table 3:** Histopathological evidence of somatosensory involvement.

**Post Mortem**			
**Author and Year of Publication**	**Study Participants**		**Significant Findings**
Mehta et al., 2021 [197]	3 with *C9orf72*-positive ALS/FTD, 2 healthy controls		- Selective dysregulation of the mitochondrially encoded transcripts in ventral horn spinal MNs, but not in corresponding dorsal horn sensory neurons
De Reuck et al., 2017 [175]	12 with ALS, 38 with FTLD, 28 healthy controls		- Iron deposition in thalamus
Fatima et al., 2015 [162]	35 with ALS, 4 healthy controls		- pTDP43 immunoreactive oligodendrocytes observed in white matter in sensory cortex
Oyanagi et al., 2015 [178]	7 with ALS		- Severe deterioration in somatosensory, auditory and gustatory pathways in brainstem and spinal cord
Rabin et al., 1999 [73]	49 with ALS		- Loss of neurons in dorsal root ganglia and degeneration of posterior columns
Shankar et al., 1995 [198]	3 with ALS		- Involvement of sensory systems
**Biopsy**			
**Author and year of publication**	**Study participants**	**Region biopsied**	**Significant findings**
Dalla Bella et al., 2016 [48]	57 with ALS	Skin	- IENF density reduced in 75.4% of pure ALS and 50% FOSMN patients - IENF density similarly reduced in different subtypes of ALS
Ruoppolo et al., 2016 [65]	114 with ALS	Larynx	- Morphological changes present in fibres in 2 patients with ALS
Truini et al., 2015 [114]	24 with ALS	Skin	- Normal intraepidermal nerve fibre
Luigetti et al., 2012 [182]	17 with ALS	Sural nerve	- More than 2/3 biopsies revealed variable degree of axonal loss
Sawa et al., 2012 [184]	3 with ALS	Sural nerve	- Moderate, marginal reduction in myelin thickness
Devigili et al., 2011 [183]	18 with ALS	Sural nerve	- 11 of 18 ALS+ patients had inflammatory cell infiltrates
Weis et al., 2011 [51]	28 with ALS	Skin	- Significant reduction in epidermal nerve fibre density in distal calf - Small-fibre neuropathy significantly higher in patients with ALS
Hammad et al., 2007 [36]	103 with ALS	Sural nerve	- Pathologic abnormalities present in 91% - Large-calibre myelinated fibres reduced in 73%, small-calibre myelinated fibres affected in 23% - Axonal degeneration and regeneration and excessive myelin irregularity
Isaacs et al., 2007 [38]	5 with ALS	Sensory nerve	- Axonal loss
Rabin et al., 1999 [73]	49 with ALS	Skin	- Intracutaneous sensory fibres in skin biopsies were normal
Hawkes et al., 1998 [59]	58 with MND, 135 healthy controls	Olfactory bulbs	- Olfactory bulbs showed excess lipofuscin deposition
Heads et al., 1991 [181]	NA	Sural nerve	- Early axonal atrophy, increased remyelination - Sensory nerve pathology in ALS was correlated with disease duration
Hamida et al., 1987 [185]	16 with ALS, 8 healthy controls	Superficial peroneal nerve	- Significant reduction in all myelinated fibres
Dyck et al., 1975 [56]	10 with ALS	Peroneal nerve	- One nerve had low myelinated fibre density, 7 of 10 have abnormally high frequencies of teased fibre abnormalities
**Animal models**			
**Author and year of publication**	**Disease model**		**Significant findings**
Baczyk et al., 2020 [199]	presymptomatic *mutSOD1* mice		- Excitatory responses evoked by sensory and brainstem inputs reduced in motoneurons of presymptomatic *mutSOD1* mice
Peng et al., 2020 [200]	Mice with astroglial TDP-43 deletion		- Mice with astroglial TDP-43 deletion develop motor but not sensory deficits
Ruiz-Soto et al., 2020 [201]	*SOD1(G93A)* mouse model		- Presymptomatic alterations of satellite glial cells (SGCs) at the dorsal root ganglion might not only be responsible of sensory disturbances in ALS, but could also contribute to anterior horn motor disturbances
Weerasekera et al., 2020 [202]	TDP-43(A315T) mouse model		- [^18^F]FDG PET demonstrated significantly lowered glucose metabolism in motor and somatosensory cortices of TDP-43A315T mice
Seki et al., 2019 [203]	*SOD1G93A* mouse		- Novel reflex circuit-specific proprioceptive sensory abnormality in ALS
Vaughan et al., 2018 [186]	*TDP43A315T* on sensory neurons in culture and in vivo, *SOD1G93A* sensory neurons		- *TDP43* sensory neurons have shorter and less complex neurites and are more sensitive to vincristine compared to controls and *SOD1* sensory neurons - Levels of ATF3 and PERK are significantly different between *TDP43* and *SOD1* sensory neurons
Marcuzzo et al., 2017 [188]	G93A-*SOD1* mouse model		- Main sensory regions affected by neurodegeneration
Bernard-Marissal et al., 2015 [196]	*SIGMAR1* mice		- Defects are not observed in cultured sensory neurons
Vaughan et al., 2015 [187]	*SOD1(G93A)* and *TDP43(A315T)*		- Degeneration of sensory nerve endings in *TDP43* mice
Sabado et al., 2014 [30]	*SOD1(G93A)* mouse model		- Large dorsal root ganglion proprioceptive neurons accumulate misfolded *SOD1* and undergo degeneration - Degenerating sensory axons were detected in association with activated microglial cells in the spinal cord dorsal horn
Cowin et al., 2011 [195]	*SOD1* mouse model		- Sensory white matter fibres unchanged
Filali et al., 2011 [189]	*SOD1(G37R)* transgenic mouse model		- Raised somatosensory thresholds
Underwood et al., 2011 [72]	*SOD1* mice		- Sensory white matter fibres preserved
Izquierdo et al., 2010 [190]	nonhuman primates		- Mediodorsal nucleus of the thalamus supports reward-based decision making
Guo et al., 2009 [192]	*hSOD1-G93A* mice		- Spinal cords of mice exhibit significant damage in the sensory system
Fischer et al., 2005 [191]	*SOD*/Wld(S) mice		- Significant degeneration of sensory axons

**Table 4:** Neuroimaging studies of ALS, highlighting somatosensory involvement.

Author and Year of Publication	Study Participants	Methodology	Significant Findings
Structural imaging			
Ahmed et al., 2021 [229]	52 with ALS, 41 with ALS-FTD, 58 with bvFTD, 58 healthy controls	Structural (volumetry)	- Marked subcortical atrophy of thalamus in ALS-FTD and bvFTD
Barry et al., 2021 [232]	12 with ALS, 9 healthy controls	Structural, MRS, rsfMRI	- Disruption in long-range functional connectivity between superior sensorimotor cortex and bilateral cerebellar lobule VI - Decreased functional connectivity that predominantly mapped to bilateral postcentral and precentral gyri
Chen et al., 2020 [246]	22 with ALS, 20 healthy controls	Structural	- Decreased fractal dimensionality (FD) values in right postcentral gyrus
Chipika et al., 2020 [207]	100 with ALS, 33 with PLS, 117 healthy controls	Structural (volumetry, shape analysis, ROI morphometry)	- Degeneration of sensory nuclei of thalamus in *C9orf72*-negative ALS and PLS
Bede et al., 2018 [32]	36 with ALS, 26 with ALS-FTD, 10 with bvFTD, 11 with nfvPPA, 5 with svPPA, 50 healthy controls	Structural (volumetry, cortical thickness), diffusion	- Patients with *C9orf72* ALS-FTD have reduced density in thalamic sub-region connected to sensory cortex
Bueno et al. 2018 [247]	20 with ALS, 15 healthy controls	Structural, diffusion, rsfMRI	- Thalamus is preserved
Chen et al., 2018 [248]	65 with ALS, 65 healthy controls	Structural	- GM volume reduced in left postcentral gyrus in spinal onset ALS
Buhour et al., 2017 [217]	37 with ALS, 37 healthy controls	Structural	- GM atrophy in right thalamus
Kim et al., 2017 [249]	62 with ALS, 57 healthy controls	Structural	- Atrophy of postcentral region in limb-onset ALS
de Albuquerque et al., 2016 [250]	32 ALS, 32 healthy controls	Structural	- Higher values for parameter correlation in both thalami
Masuda et al., 2016 [219]	44 with ALS, 7 with ALS-FTD, 24 healthy controls	Structural (VBM), diffusion	- Atrophic changes in thalamus in ALS-FTD
Devine et al., 2015 [45]	30 with ALS, 17 healthy controls	Structural	- Asymmetric atrophy of the left somatosensory cortex in ALS patients with right-sided onset of limb weakness
Machts et al., 2015 [209]	42 with ALS-nci, 7 with ALS-FTD, 18 with ALS-plus, 39 healthy controls	Structural (volumetry, shape, density)	- Pathologic changes in bilateral thalami
Irwin et al., 2013 [251]	143 ALS	structural	- Greater atrophy in thalamus
Mioshi et al., 2013 [252]	22 with ALS, 17 with ALS-FTD, 18 healthy controls	Structural (VBM)	- Atrophy in somatosensory region in ALS-plus
Thorns et al., 2013 [253]	14 with ALS, 14 healthy controls	Structural (cortical thickness)	- Significant cortical thinning in postcentral gyrus bilaterally
Cosottini et al., 2012 [230]	20 with ALS, 16 healthy controls	Structural (VBM), fMRI	- Decreased cortical GM in postcentral gyri - Significant hypoactivation of primary sensory motor cortex
Mohammadi et al., 2009 [254]	20 with ALS, 20 healthy controls	Structural, fMRI	- ALS patients without bulbar involvement show activations in postcentral areas and thalamus
Grosskreutz et al., 2006 [231]	17 with ALS, 17 healthy controls	Structural	- Decreased GM volume in postcentral gyrus bilaterally
Chang et al., 2005 [228]	10 with ALS, 10 with ALS-FTD, 22 healthy controls	Structural (VBM)	- GM atrophy in left posterior thalamus
Kato et al., 1993 [255]	22 with ALS	Structural	- Gradually progressive atrophy in postcentral gyrus - High intensity T2 signals rarely in the thalamus
Diffusion imaging			
Rajagopalan et al., 2021 [256]	75 with ALS, 14 disease controls	diffusion	- ALS-FTD showed significantly lower primary motor and sensory cortex GM fractal dimension values compared to other ALS groups
Rajagopalan et al., 2017 [257]	45 with ALS, 14 healthy controls	Diffusion	- Fibres projecting to postcentral gyrus were spared
Zhang et al., 2017 [218]	38 with ALS, 35 healthy controls	Diffusion	- Thalamocortical connections remained relatively in tact
Sheelakumari et al., 2016 [258]	17 with ALS, 15 healthy controls	Diffusion, SWI	- No significant differences in sensory cortex
Trojsi et al., 2015 [259]	54 with ALS, 18 healthy controls	Diffusion	- Reduced FA and increased RD and MD in WM underneath postcentral gyri
Barbagallo et al., 2014 [221]	24 with ALS, 22 healthy controls	Diffusion, structural	- MD values of ALS significantly higher in thalamus
Kim et al., 2014 [260]	14 with ALS, 16 healthy controls	Diffusion	- No significant changes in primary sensory cortex
Sharma et al., 2013 [227]	14 with ALS, 12 healthy controls	Diffusion, structural	- MD significantly higher in thalamus
Rose et al., 2012 [237]	15 with ALS, 20 healthy controls	Diffusion, structural	- Abnormal intrahemispheric pathways include CST involving right postcentral gyrus
Agosta et al., 2011 [261]	26 with ALS, 15 healthy controls	Diffusion	- Significantly increased functional connectivity between left sensorimotor cortex and other cortical areas and cerebellum - Different changes in functional connectivity in patients with CST damage versus those without
Thivard et al., 2007 [224]	15 with ALS, 25 healthy controls	Diffusion, structural	- Decreased FA in thalamus
Sach et al., 2004 [226]	15 with ALS, 12 healthy controls	Diffusion, structural,	- Decreased FA in thalamus
Functional imaging			
Wei et al., 2021 [262]	20 with ALS, 22 healthy controls	fMRI	- ALS group had significantly increased functional stability in postcentral gyrus
Qiu et al., 2019 [263]	60 with ALS, 60 healthy controls	rsfMRI, structural, diffusion	- Reduced functional connectivity in bilateral postcentral gyrus
Menke et al., 2017 [215]	13 with ALS, 3 with PLS	rsfMRI, structural (volumetry, shape analysis), diffusion	- Reduced functional connectivity between sensorimotor resting state network and frontal pole
Xu et al., 2017 [216]	20 with ALS, 21 healthy controls	rsfMRI	- Decreased nodal efficiency in right thalamus
Zhang et al., 2017 [264]	38 with ALS, 35 healthy controls	rsfMRI, diffusion	- Reduced voxel mirrored homotopic connectivity in postcentral gyrus
Fang et al., 2016 [245]	20 with ALS, 21 healthy controls	rsfMRI	- Significant regional activity alterations in left primary somatosensory cortex
Zhou et al., 2016 [44]	43 with ALS, 44 healthy controls	rsfMRI	- Significant decrease in DC in bilateral sensory motor region
Zhou et al., 2014 [236]	12 with ALS, 12 healthy controls	rsfMRI	- Decreased coherence in superior medial sensory-motor network (SMN) - Increased coherence in peripheral SMN areas - Decreased regional coherence in right postcentral gyrus is correlated with high disease severity - Enhanced regional coherence in left postcentral gyrus is related to longer disease duration - Increased coherence in left postcentral gyrus corresponds to fast disease progression rate
Poujois et al., 2013 [265]	19 with ALS, 21 healthy controls	fMRI, diffusion	- Overactivations in ipsilateral and contralateral somatosensory cortex - Correlation of ipsilateral somatosensory activations with severity of right arm deficit
Luo et al., 2012 [238]	20 with ALS, 20 healthy controls	fMRI, structural (VBM)	- Significant reduction in ALFF in right postcentral gyrus
Mohammadi et al., 2011 [266]	22 with ALS, 22 healthy controls	fMRI	- Size of activated area in contralateral sensorimotor cortex increased to similar degree in all 3 ALS groups compared to controls regardless of weakness in clinical examination
Lule et al., 2010 [46]	14 with ALS, 18 healthy controls	fMRI	- Reduced response in sensory integration areas of parietal lobe
Li et al., 2009 [223]	10 with ALS, 10 healthy controls	fMRI, diffusion, structural	- Reduced activation of sensorimotor cortex in patients with dysphagia - MD increased in thalamus
Other			
De Reuck et al., 2017 [175]	12 with ALS, 38 with FTLD, 28 healthy controls	Iron deposition	- Significant increase of iron deposition in thalamus
Pioro et al., 1994 [235]	12 with MND, 6 healthy controls	1H-MRSI	- Significant decrease in Na/Cr resonance intensity ratios in primary sensory region
Spinal imaging			
Pisharady et al., 2020 [267]	20 with ALS, 20 healthy controls	Diffusion	- Tracts and spinal levels affected in ALS, involvement of sensory pathways
Olney et al., 2018 [268]	3 with ALS, 2 with ALS-FTD, 1 with PLS, 2 with PMA, 2 with FOSMN, 10 healthy controls	Structural-axial 2D PSIR images and diffusion	- Spinal cord GM and WM atrophy
Rasoanandrianina et al., 2017 [206]	10 with ALS, 20 healthy controls	Diffusion, ihMT	- Changes in posterior spinocerebellar tract
Wang et al., 2017 [269]	14 with ALS, 14 healthy controls	MRS	- Significantly decreased Naa/Cr ratios in postcentral gyrus
Iglesias et al., 2015 [41]	21 with ALS, 21 healthy controls	Diffusion	- Damage to ascending sensory fibres in ~60% of patients
Wang et al., 2014 [270]	24 with ALS, 16 healthy controls	Structural, diffusion	- No abnormal findings in cervical spine detected with conventional MR imaging
Cohen-Adad et al., 2013 [179]	29 with ALS, 21 healthy controls	Structural, diffusion, magnetization transfer	- Impairment of spinal sensory pathways detected in early-stage disease

## Glossary

AI—artificial intelligence; ALFF—amplitude of low-frequency fluctuations; ALS—amyotrophic lateral sclerosis; ALSbi—ALS with behavioural impairment; ALSci—ALS with cognitive impairment; ALSFRS-R—Amyotrophic Lateral Sclerosis Functional Rating Scale; ALS-FTD—amyotrophic lateral sclerosis with frontotemporal degeneration; ASO—antisense oligonucleotide; ATF3—AMP-dependent transcription factor; BAEP—brainstem auditory evoked potential; bvFTD—behavioural variant frontotemporal dementia; C9orf72—chromosome 9 open reading frame 72; CMAP—compound muscle action potential; CPM—conditioned pain modulation; CSAP—compound sensory action potential; CST—corticospinal tract; CV—conduction velocity; DC—degree centrality; DRG—dorsal root ganglion; DTI—diffusion tensor imaging; EMG—electromyography; ENG—electronystagmography; Eps—evoked potentials; FA—fractional anisotropy; fALFF—fractional amplitude of low-frequency fluctuations; fALS—familial ALS; FD—fractal dimensionality; FDA—Food and Drug Administration; FDG-PET—fluorodeoxyglucose (FDG)-positron emission tomography; FEESST—fibre-optic endoscopic evaluation of swallowing with sensory testing; fMRI—functional MRI; FOSMN—facial onset sensory and motor neuronopathy; FTD—frontotemporal degeneration; FTLD—frontotemporal lobar degeneration; GM—grey matter; HSP—hereditary spastic paraplegia; IENFD—intraepidermal nerve fibre density; ihMT—inhomogeneous magnetization transfer; LEP—laser evoked potentials; MCV—motor conduction velocity; MD—mean diffusivity; ML—machine learning; MND—motor neuron disease; MRI—magnetic resonance imaging; MRS—magnetic resonance spectroscopy; MUNE—motor unit number estimation; Na/Cr—N-acetyl to creatine resonance intensity ratios; NCS—nerve conduction study; NCV—nerve conduction velocity; NHPT—nine-hole peg test; NMS—non-motor symptoms; OCT—optical coherence tomography; OEP—olfactory-evoked response; PERK—PKR-like endoplasmic Reticulum Kinase; PLS—primary lateral sclerosis; PMA—progressive muscular atrophy; PPS—post-poliomyelitis syndrome; PRISMA—Preferred Reporting Items for Systematic Reviews and Meta-Analyses; PSVEP—pattern-shift visual evoked response; QoL—quality of life; QST—quantitative sensory testing; RD—radial diffusivity; ROI—region of interest; rsfMRI—resting state functional MRI; SAPAs—sensory action potential amplitudes; SBMA—spinal and bulbar muscular atrophy (Kennedy’s disease); SCNV—sensory nerve conduction velocity; SCV—sensory conduction velocity; SEP—somatosensory evoked potentials; SGCs—satellite glial cells; SMN—superior medial sensory-motor network; SNAP—sensory nerve action potential; SNAPA—sensory nerve action potential amplitude; SOD1—superoxide dismutase 1; SSCV—spinal cord conduction velocity; SSEP—somatosensory evoked potentials; TDP-43—TAR DNA-binding protein 43; UPSIT—University of Pennsylvania Smell Identification Test; UtC—urge to cough; VBM—voxel-based morphometry; VEP—visual evoked potential; WM—white matter.
